# Whatever happened to *Bishopanthus* (Compositae, Liabeae)?

**DOI:** 10.3897/phytokeys.30.6652

**Published:** 2013-12-04

**Authors:** Vicki A. Funk, Harold Robinson, Alice R. Tangerini

**Affiliations:** 1 Department of Botany, MRC 166, National Museum of Natural History, P.O. Box 37012, Smithsonian Institution, Washington DC 20013-7012

**Keywords:** Asteraceae, *Bishopanthus*, Compositae, Liabeae, Neotropics, Peru

## Abstract

The enigmatic monospecific *Bishopanthus* of the tribe Liabeae (Compostiae/Asteraceae) has never been fully understood. It has not been possible to examine it in detail since it was described, because most of the type material was destroyed shortly after it arrived at the US National Herbarium, the morphology is insufficient to assign it to a subtribe, and the small amount of leaf material that remains is unsuitable for DNA extraction. A detailed description in English, image of the type specimen, photograph, and original illustration are included along with an estimation of where it was collected in the hopes that this information will encourage other field botanists who collect in northern Peru to search for it.

## Introduction

Robert Merrill King (1930–2007) was associated with the Smithsonian Institution from 1962 until 1998. During that time he organized many collecting trips to gather Compositae, mostly in the Neotropics. Among the most successful trips made by King, were those with Luther Earl Bishop (1943–1993). Thus, it was during a trip to Peru in early 1983 that Bishop, who frequently left the roadside and hiked into nearby areas, walked up a trail beside a small shop with hanging potted plants and returned within a half hour with a plant that has caused us problems for 30 years.

*Problem 1)* The collection arrived at the US National Herbarium (Smithsonian Institution) only a short time before the publication of a monograph by Robinson (1983a) that was supposed to be a complete summary of the Asteraceous tribe Liabeae. The collection was immediately recognized as a new genus (by HR) and, in effect, it made the monograph he had completed obsolete before it was published. *Bishopanthus* was published later that year in *Phytologia* (Robinson 1983b).

*Problem 2)* The original five ample sheets of the gathering were destroyed except for a few small scraps hidden under a bookcase that ultimately became the type.

*Problem 3)* Attempts by Michael Dillon (F) to collect the plant, based on information from King provided to Robinson, failed to find any sign of the species.

*Problem 4)* The locality data were skimpy and the original collector, Bishop, and the leader of the expedition, King, have since died, so it is not possible to obtain any additional information.

*Problem 5)* Morphological analyses have failed to definitively place the species in a subtribe (Funk et al. 2012). Some of the characters link it to the Liabinae and others to a more remote position, as yet undefined. As a result, the genus is currently designated *insertae sedis*.

*Problem 6)* Dried leaf material that was collected for chemical analysis (Singh et al. 1985) was apparently discarded after the retirement of both Bohlmann & Jakupovic.

*Problem 7)* During the King & Bishop expeditions the herbarium material was placed in alcohol before being dried and so it is not suitable for current methods of DNA analysis.

**Figure 1.**
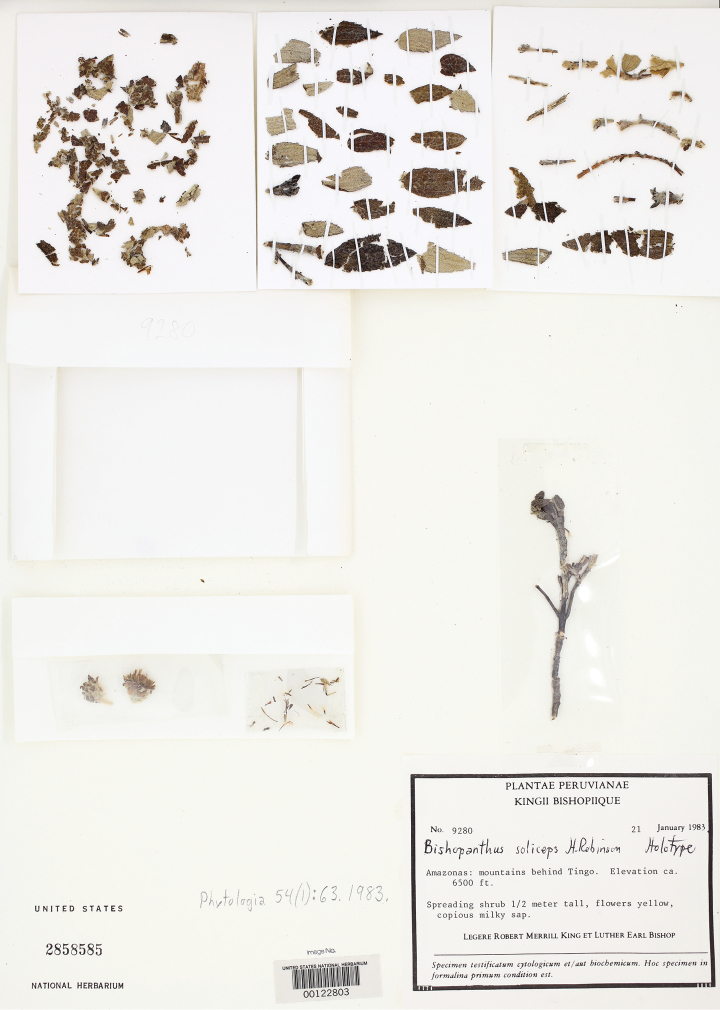
Image of the holotype of *Bishopanthus soliceps* H. Rob. from the US National Herbarium (US).

**Figure 2.**
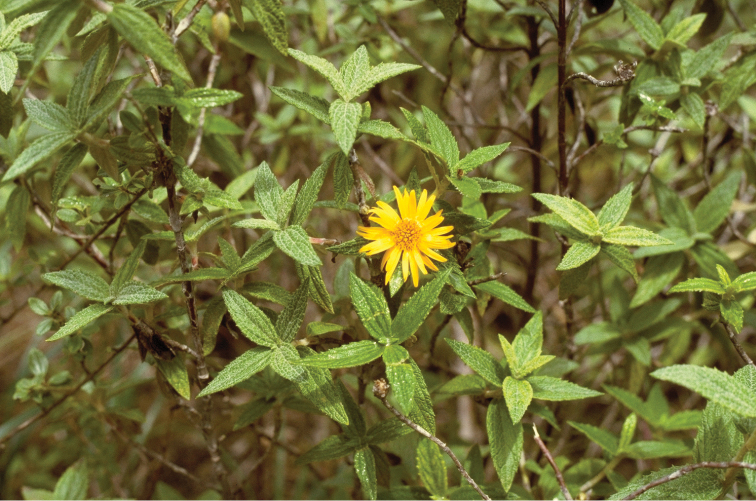
Field photograph of *Bishopanthus soliceps* H. Rob. by L. E. Bishop for whom the genus was named.

**Figure 3.**
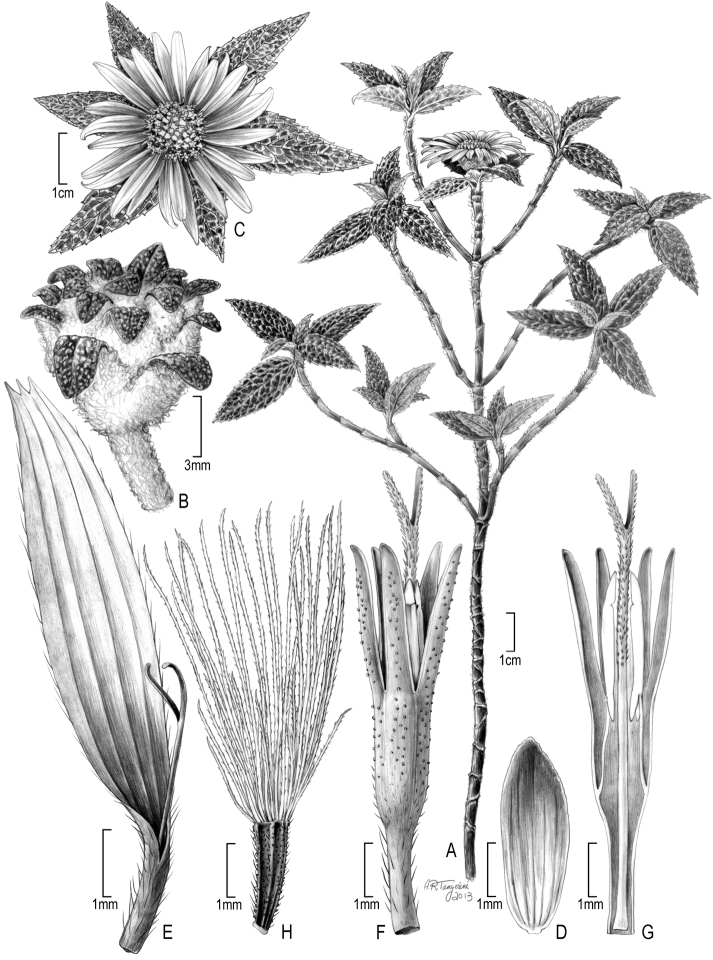
Illustration of *Bishopanthus soliceps* H. Rob.: **A** habit **B** head in pre-flowering stage **C** flowering head from above **D** involucral bract **E** ray flower **F** disc flower **G** longitudinal view of disc flower **H** achene with pappus. [Illustration by Alice Tangerini]

In summary, what survives of the species is the herbarium specimen that has only the small scraps that became the type (Fig. 1), a color photograph taken by Earl Bishop (Fig. 2; Funk et al. 1996: 550, in black and white), a microscope slide prepared by Robinson from material before the specimens were destroyed (remade from the original Hoyer’s solution slide during this study), the results of the chemical analysis by Singh et al. (1985) from a sample sent to him by R.M. King, and a picture of the pollen published in the pollen study of the Liabeae (Robinson and Marticorena 1986). From this material we have reconstructed the plant and an illustration has been produced (Fig. 3).

## Materials and methods

The dried specimen material of *Bishopanthus* (what remained of the type specimen after it was destroyed) consisted of small fragments of leaves, a few small stems, an immature head and one mature head with three broken ray flowers (Fig. 1). The disk flowers faired better because they were protected if somewhat compressed. Additional material consisted of one slide of the dissected disk flowers with a complete ray and a sectioned disk flower showing the anthers and style. From this material Tangerini was able to piece together the leaf fragments to form a few almost whole leaves and mounted these and other fragments and stems on two archival 4” by 5” cards (Fig. 1). Tiny mylar packets were made to hold the two heads and dissected disk flowers. Drawings of all of the floral details were made using a combination of the dried and slide material and the habit was reconstructed from the single photograph which provided an invaluable reference for the structure of the habit and leaf insertion. Conversations with Robinson (who had seen all of the original five duplicates) provided information on the flower position on the stem. The final drawing was done with a variety of polycarbonate pencils (Turquoise Filmograph, Mars Duralar and Mars Dynagraph, and L. & C. Hardtmuth Koh-I-Lar pencils) on Grafix double matte drafting film. These pencils produce a denser black than regular graphite pencils and the subtle gray tones give the impression of the bullate leaf surface and the woolly texture of the involucres. Shading is applied with the softer leads first followed by the harder leads to add fine lines and smaller details. The polycarbonate leads are waxier and do not smudge as much as graphite. Unfortunately the polycarbonate leads went out of production in the mid 1990’s so this technique has a limited lifetime dependent on acquisition of the remaining stock of pencils. Some botanical art schools have supplies for illustration classes and perhaps resurgence in interest will spur new production.

The microscope slide was made using Hoyer’s solution with a formula as given by Anderson (1954): Distilled water 50 cc; Gum arabic (U.S.P. Flake) 30 grams; Chloral hydrate 200 grams; Glycerin 20 cc. Since the original application of this method, Chloral hydrate has become a controlled substance, which limits access. More important is the impermanent nature of Hoyer’s slides unless the cover slips are ringed with waterproof sealant (e.g., clear fingernail polish). The fact that microscope slides in Hoyer’s solution can dry up does not mean they should be discarded. The water miscible solution that makes the medium so useful as a rapid mounting medium also allows for recovery of any material on old dried slides. This should be noted by anyone inclined to discard old slides that often contain valuable type material.

Until this paper, *Bishopanthus* was the only genus of the Liabeae that had not been illustrated by Tangerini using the method described above (Robinson 1983a, Funk and Robinson 2001, 2009, Robinson and Funk 2011). Now that we have this final drawing it is possible to look at all of the genera and compare the morphology.

## Systematics

### Bishopanthus

H. Rob., Phytologia 54(1): 63. 1983.

http://species-id.net/wiki/Bishopanthus

http://www.biodiversitylibrary.org/item/46790#page/71/mode/1up

#### Type species.

*Bishopanthus soliceps* H. Rob.

#### Type.

Peru; Amazonas, mountains behind Tingo, 6500 Ft., 21 Jan 1983, *R.M. King & L.E. Bishop 9280* (holotype: US).

#### Description.

*Shrubs* to 0.5 m tall, moderately to multi branched; *stems* with latex, pale reddish, internodes short, jointed, densely white-wooly strongly invested by leaf bases. *Leaves* opposite, bases strongly vaginate, vagination usually ca. 5 mm long, longer than the internode, imbricate parts of vaginate bases wooly-tomentose externally; *petioles* short, ca. 0.5 mm long; *blades* oblong-ovate, mostly 2–4 cm long and 8–16 mm wide, base rounded, margins with many distinct small teeth, apex shortly acute, sub-longitudinally trinervate from near base, upper surface bullate with bulging areoles, major nerves sunken and diffusely arachnoid-tomentose, undersurface densely, grayish-wooly tomentose, major veins raised. *Inflorescence* abruptly terminal on leafy branches. *Heads* solitary, over-topped by lateral branches, ca. 10 mm high, ca. 12 mm wide, excluding rays; *involucral bracts* subequal, ca. 25, in ca. 2 series, oblong-lanceolate, 7–8 mm long and ca. 1.5 mm wide, outer bracts with apices reflexed, outer surface distally green, subglabrous, below densely white wooly-tomentose, inner bracts not reflexed distally, acute, subglabrous. *Ray flowers* ca. 20, female; corollas yellow, basal tube 2.5–3.5 mm long, narrowly funnelform with sparse spreading trichomes; *limbs* linear, 11–12 mm long, to 2 mm wide, apices tridentate, basally with minute, short biseriate trichomes, distally rather densely arachnoid-tomentose and gland-dotted. *Disc flowers* ca. 25, bisexual; *corollas* yellow, 7.0–7.5 mm long, basal tube ca. 2.5 mm long, hirtellous with sparse straight-spreading trichomes, trichomes with one row of cells, throat ca. 2.5 mm long, subcylindrical, below with few short biseriate trichomes and fewer uniseriate trichomes, distally with almost no trichomes and sparsely gland-dotted, lobes linear, ca. 2.8 mm long and 0.5 mm wide, near margins distally with few stomata and many glandular dots, rather densely arachnoid-tomentose. *Anther* filament collars ca. 0.25 mm long, with cells shortly oblong, cell walls firm, in-ornate; *thecae* ca. 2.5 mm long, endothecial cells obscure, somewhat oblong, shields tenuously irregularly areolate; *apical appendage* oblong-ovate, ca. 0.4–0.5 mm long, 0.22 mm wide, glabrous. *Style* base with distinct expanded node, hispidulous upper part of style shaft ca. 3 mm long; *branches* ca. 1 mm long. *Achenes* ca. 2.7 mm long, 8–10-ribbed, with short trichomes, setulae and glands, setulae numerous, contorted, distally on achenes longer, trichomes very sparse, with single row of cells, glands sparse, short-stipitate, with minute capitula; *carpopodia* shortly stopper-shaped, sub-annuliform, ca. 0.35 mm wide, 0.15 mm high; with cells in 12–15 series, 12–15 μm in diameter, with thickened walls. *Pappus* setae densely congested, larger setae ca. 35, sometimes irregularly elongate, mostly 4.5–6.0 mm long, apices tenuous, outer series of setae shorter, narrower, mostly 0.7–1.0 mm long, scabrous, simple. *Pollen* ca. 37μm in diameter, irregularly spinulose.

#### Diagnosis.

Small shrub with milky sap; opposite leaves that are tri-nervate with a bullate upper surface and an under surface covered with a dense, grayish-wooly tomentum; two rows of subequal involucral bracts; short (~ 1mm) style branches; 8–10 ribs on the achenes and a bi-seriate pappus of subequal bristles (Table 1).

**Table 1.** Diagnostic characters of the genera of the Liabeae. Note the dual placement of *Bishopanthus*.

**Genus**	**Habit**	**latex**	**veination**	**bullate lvs**	**under surface**	**Ray Fls**	**anther color**	**Pollen spines**	**ray fl**	**style**	**achene**	**inner pappus**	**outer pappus**
*Cacosmia*	Erect shrubs	yes	3	yes	wooly	yes	light yellow	irregular	yellow	short	4-5 angled	none	none
***Bishopanthus***	**Shrub 0.5 m**	**yes**	**3**	**yes**	**wooly**	**yes**	**light yellow**	**irregular**	**yellow**	**short**	**8 to 10 ribs**	**bristles**	**bristles**
*Ferreyranthus*	Shrub-sm trees	no	pinnate	yes	wooly	yes	light yellow	irregular	yellow	long	~10 ribs	bristles	scales
*Oligactis*	Shrubs/vines	no	pinnate	yes	wooly	yes	light yellow	irregular	yellow	long	5 to 8 ribs	bristles	scales/ bristles
*Dillandia*	Herbs	no	pinnate	yes	wooly	yes	light yellow	irregular	yellow	long	7 to 10 ribs	bristles	lost
*Sampera*	Shrubs	no	pinnate	yes/no	wooly	yes	light yellow	irregular	yellow	long	5 to 8 ribs	bristles	scales
*Liabum*	Perennial herbs/subshrubs	no	pinnate/ weakly 3	no	wooly	yes	light yellow	irregular	yellow	long	10 ribs	bristles	bristles
*Sinclairia*	perennial herbs/woody vines/subshrubs/sm trees	yes	3	no	wooly	yes & no	light yellow	irregular	yellow	long	8 to 10 ribs	bristles	scales
*Sinclairiopsis*	Shrub	yes	3	no	wooly	yes	light yellow	irregular	yellow	long	~10 ribs	bristles	lost
*Munnozia* 1	Shrubs	yes	3	no	wooly	yes	dark	regular	white	short	6 to 10 ribs	bristles	scales
*Chrysactinium*	Perennial herbs	yes	3	no	wooly	yes	dark	regular	yellow	short	~10 ribs	bristles	none
*Munnozia* 2 & 3	Annual/perennial herbs; shrubs/subshrubs	yes	3	no	wooly	yes	dark	regular	yellow	short	6 to 10 ribs	bristles	scales
*Chionopappus*	Erect shrubs	yes	3	no	wooly	yes	light yellow	irregular	red	short	8 to 10 ribs	plumose	none
*Erato*	Perennial herbs/subshrubs	yes	palmate	no	glabrous	yes	dark	irregular	yellow	short	4-sided	bristles	none
*Philoglossa*	Decumbent or creeping herbs	yes	3	no	glabrous	yes	dark	irregular	yellow	short	2-compressed	none	none
*Paranephelius*	Perennial herbs	yes	pinnate	yes/no	wooly	yes	light yellow	irregular	yellow	short	~10 ribs	bristles	bristles
*Pseudonoseris*	Perennial herbs	yes	pinnate	yes/no	wooly	yes	light yellow	irregular	red	short	~10 ribs	bristles	scales
*Bishopanthus*	Shrub 0.5 m	yes	3	yes	wooly	yes	light yellow	irregular	yellow	short	8 to 10 ribs	bristles	bristles
*Stephanbeckia*	Perennial herbs	yes	3	no	wooly	yes	light yellow	irregular	yellow	short	2-compressed	plumose	none
*Microliabum*	Annual-perennial herb/ subshrub	yes	3	no	wooly	yes	light yellow	irregular	yellow	short	8 to 10 ribs	scales/bristles	scales/bristles
***Bishopanthus***	**Shrub 0.5 m**	**yes**	**3**	**yes**	**wooly**	**yes**	**light yellow**	**irregular**	**yellow**	**short**	**8 to 10 ribs**	**bristles**	**bristles**

All members of the Liabeae have opposite leaves although some have very short internodes so they appear to have rosettes. Also, all members have milky sap and/or wooly light colored tomentum on the undersurface of the leaves.

The specimen label says that the plants are ½ m tall, have yellow flowers and lots of milky sap. An examination of the field notebook from the King & Bishop expedition did not provide any additional information about the plant or the location, however, an examination of their route that day shows that on the 21st of January their collecting started as they drove south from Tingo (6°22'18.14"S, 77°54'38.54"W). Their first stop was 3 km south of Tingo and they proceeded south stopping occasionally until they reached their fifth stop at 31 kms south of Tingo, probably near the village of Yerbabuena (6°33'57.92"S, 77°49'49.35W). The first five stops are all listed as ca. 5500 ft in elevation (based on Google Earth this seems a bit low) but with different kilometer distances. The next stop (# 6) is where they collected *Bishopanthus*, however at this point they stopped giving the kilometers and just said “mountains behind Tingo ca. 6500 ft"and it is the only collection at that stop. The final stop of the day (# 7) has the same location with an elevation of 7000 ft. At the 7^th^ stop they collected *Cronquistianthus bishopii* King & H. Rob., also restricted to this area. The next day (22 January 1983) the first collecting stop was 61 kms along the road from Chachapoyas NW of Jaen. Evidently they were working out of Chachapoyas. We think that on the 21st of January they turned around at the 5th stop (31 km) and headed back to Chachapoyas, passed through Tingo, and stopped somewhere on the more eastern road between Tingo and Chachapoyas at 6500 Ft. The elevation on this road reaches 9000 ft. According to Google Earth the elevation is just about correct at the following coordinates: 6°21'40"S; 77°54'4"W. Of course this is a guess because the real location has never been found.

## Conclusion

In their 2012 paper Funk et al. suggested that the shrubby habit and bullate leaves of *Bishopanthus* were similar to the taxa found in the basal grade of the subtribe Liabinae (e.g., *Ferreyranthus*), but those genera lack latex and have pinnate venation and long style branches, in contrast to *Bishopanthus* (Table 1). *Bishopanthus* shares a number of characters with *Cacosmia* which is sometimes placed as the sister group of the Liabinae (Table 1). But *Cacosmia* has lost its pappus and has a unique (to Liabeae) achene (Table 1; first placement). In addition, *Cacosmia* is unique in the tribe in that it has a small cylindrical head (5 ray flowers and 5–6 disc flowers) with a highly imbricate involucre (5 series and mostly ranked) while *Bishopanthus* has a broadly campanulate head (like most of the other genera in the tribe) that is larger (20/25) and an involucre of two subequal series; it also has a very different habit. Now that we have the additional information on *Bishopanthus*, it looks as though it may be related to the *Austroliabum* element of *Microliabum* (Table 1; second placement) except that it does not have bullate leaves. Such a relationship would place it in the Paranepheliinae subtribe. Typical *Austroliabum* has been placed with some doubt as a synonym of Microliabum. Two of the typical species of *Microliabum* have been sequenced and now we need fresh material of *Bishopanthus* so we can see if either of our predictions is correct.

## Supplementary Material

XML Treatment for Bishopanthus
